# The predictive value of the *in vitro* platelet toxicity assay (iPTA) for the diagnosis of hypersensitivity reactions to sulfonamides: a case-control study

**DOI:** 10.1186/2045-7022-4-S3-P27

**Published:** 2014-07-18

**Authors:** Abdelbaset Elzagallaai, Gideon Koren, Michael Rieder

**Affiliations:** 1 Western University, Canada; 2 Western University, Physiology and Pharmacology, Canada; 3 Western University, Paediatrics, Canada

## Background

Drug hypersensitivity reactions (DHRs) are rare but potentially fatal types of adverse drug reactions (ADRs) that develop in susceptible patients following exposure to certain drugs including sulfonamides. The diagnosis of this type of ADRs is challenging and currently depends on clinical expertise. A safe and reliable *in vitro* test to diagnose DHRs would be a major advance in patient care and in evaluation of possible serious ADRs during drug development and clinical trials. Current available *in vitro* tests including the lymphocyte toxicity assay (LTA) and the lymphocyte transformation test (LTT) are cumbersome and expensive, and their predictive values are undefined. We have recently developed a novel *in vitro* diagnostic test for DHRs, the *in vitro* platelet toxicity assay (iPTA) to test patient susceptibility to DHS. The aim of this study was to evaluate the predictive value of the iPTA in diagnosis of DHRs to sulfonamide antibiotics.

## Method

We have recruited 66 individuals (36 DHS-sulfa patients and 30 healthy controls) to participate in the study. The DHR cases were referred and diagnosed based on rigorous internationally accepted criteria. Blood samples were obtained and both LTA and iPTA were performed independently. Results were then analyzed to determine the degree of agreement between the likelihood of the occurrence of the reaction as determined clinically and the performance of the two diagnostic approaches.

## Results

There was concentration-dependent toxicity in the cells of patients when incubated with the reactive hydroxylamine metabolite of sulfamethoxazole for both the LTA and iPTA (p<0.05) and toxicity was significantly greater for the cells of patients versus controls (p<0.05). The tests had a high degree of agreement (correlation coefficient: R2 = 0.97). The iPTA was significantly more sensitive than the conventional LTA test in detecting the susceptibility of patient cells to *in vitro* toxicity (p<0.05).

## Conclusion

The novel iPTA has considerable potential as an investigative tool for DHS as it is cheaper to perform and requires no special reagents that make it an excellent candidate for wider use. The iPTA has also a greater sensitivity in detecting patients with predisposition to develop DHRs to sulfonamides and other drugs and thus can be used to screen for susceptible patients prior to drug prescribing and during drug development process.

